# Adult Intussusception and Ischemic Bowel Potentially Associated with CurQD Supplementation: A Case Report of A Diagnosis Driven by Point-of-care Ultrasound

**DOI:** 10.5811/cpcem.61903

**Published:** 2026-07-10

**Authors:** Andrew Parambath, Bobby Patel, Timothy J. Batchelor, Nicholas Geoffrey Ashenburg, Terence Lee Ahern

**Affiliations:** Stanford University School of Medicine, Department of Emergency Medicine, Palo Alto, California

**Keywords:** intussusception, point-of-care ultrasound, ileocolic, ulcerative colitis, curcumin-qing dai (CurQD)

## Abstract

**Introduction:**

Adult ileocolic intussusception is rare and typically associated with a pathological lead point. Diagnosis can be challenging in the emergency department (ED), particularly when initial vital signs and laboratory studies are reassuring. Point-of-care ultrasound (POCUS) may allow for earlier recognition and expedited management.

**Case Report:**

A woman with ulcerative colitis presented to the ED with abrupt, severe, waxing-and-waning abdominal pain. Initial vital signs and lab studies were normal. Bedside POCUS performed at the point of maximal tenderness revealed a target sign in the right mid-abdomen, prompting concern for intussusception. This finding was used to advocate for urgent surgical evaluation and expedited computed tomography of the abdomen and pelvis, which confirmed ileocolic intussusception with early distal small-bowel obstruction and no identifiable mass. Despite reassuring objective data, the patient’s persistent severe pain and serial examinations raised concern for evolving ischemia. Following continued emergency physician advocacy, the patient was admitted for operative management. Diagnostic laparoscopy was converted to open laparotomy, during which manual reduction and right hemicolectomy were performed for an ischemic, near-perforated colon, followed by creation of an end ileostomy and mucus fistula. The patient recovered well postoperatively. She had been taking curcumin–qing dai (CurQD) for ulcerative colitis, a supplement with rarely reported associations with intussusception.

**Conclusion:**

This case highlights the value of emergency POCUS in identifying high-risk abdominal pathology, the importance of early surgical involvement despite reassuring initial tests, and a potential supplement-associated risk factor in patients with inflammatory bowel disease.

## INTRODUCTION

Adult intussusception represents 1%–5% of all cases of bowel obstruction and differs from pediatric forms in its etiology and management.^1^–^2^ In adults, a pathological lead point, such as a benign or malignant neoplasm, is identified in up to 90% of cases.^3^–^4^ Historically, delayed diagnosis has been common due to nonspecific symptoms and the intermittent nature of the pain.^5^ Recent advances in imaging, particularly the use of ultrasound in the emergency department (ED), allow for earlier identification and expedited surgical consultation.^6^–^8^ Because clinicians can set up point-of-care ultrasound (POCUS) quickly, deploy it at the bedside, and capture transient episodes of intussusception, POCUS may uniquely identify pathology that resolves before formal imaging occurs. Clinicians can visualize characteristic findings, such as the target and pseudokidney signs, and increasingly use POCUS as an adjunct to computed tomography (CT) when evaluating acute abdominal pain.^9^–^10^ This report highlights a case in which bedside ultrasound findings enabled the emergency physician to advocate for urgent surgical intervention in a patient with ulcerative colitis and normal laboratory values, preventing bowel perforation and additional morbidity.

## CASE REPORT

A 29-year-old woman with ulcerative colitis and celiac disease developed acute severe abdominal pain at approximately 4 pm while at work. Pain was constant with waxing and waning intensity ranging from 5/10 to 10/10, predominantly in the upper abdomen, worse on the right, and radiating posteriorly. She denied vomiting, diarrhea, fever, chills, and vaginal discharge, and had not eaten since symptom onset. Current medications included a prednisone taper for a recent ulcerative colitis flare, nitrofurantoin for urinary tract infection, and an over-the-counter curcumin-qing dai (CurQD) supplement for colitis. Of note, CurQD listed intussusception as a potential side effect on its supplement labeling.^11^–^12^

On examination, she was afebrile with normal vital signs. The abdomen was soft but tender to palpation in the right greater than left quadrants, with intermittent guarding during pain spikes but no rebound or peritoneal signs. There was no pulsatile mass. The initial differential diagnosis included biliary colic, cholecystitis, appendicitis, small-bowel obstruction, intussusception, ovarian torsion, and mesenteric ischemia. Given the patient’s waxing and waning pain, absence of peritoneal signs, and history of inflammatory bowel disease, intussusception was considered possible.

**Image 1:**
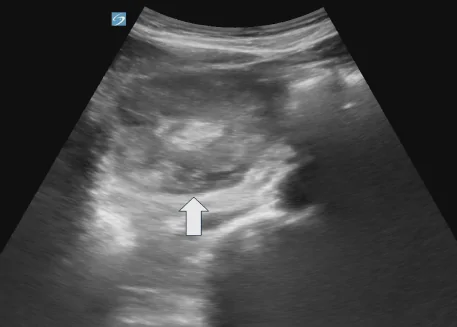
Point-of-care ultrasound (transverse view, right mid-abdomen) demonstrating the classic “target” sign consistent with ileocolic intussusception. Concentric hypoechoic and hyperechoic rings represent the bowel-within-bowel configuration.

A focused abdominal POCUS performed at the point of maximal tenderness demonstrated a concentric “target” configuration in the right mid-abdomen, concerning for intussusception (Image 1). The POCUS finding became the critical evidence used to advocate for surgical consultation despite normal lab values and stable vital signs.

**Image 2:**
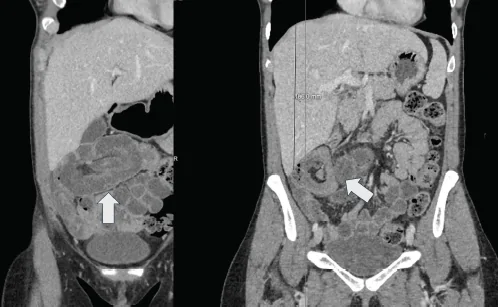
Contrast-enhanced computed tomography abdomen and pelvis (coronal reformatted images) demonstrating ileocolic intussusception (arrows) with characteristic “bowel-within-bowel” configuration. Mesenteric fat and vessels were dragged into the intussusceptum, producing a layered appearance consistent with the target sign. Findings indicate early small-bowel obstruction without pneumatosis or perforation.

The emergency physician expedited a contrast-enhanced computed tomography (CT) abdomen/pelvis. Radiology reported ileocolic intussusception with suspected early distal small-bowel obstruction, no appreciable mass to suggest a lead point, and no definitive ischemia or perforation.^13^ An incidental finding suggested compression of the left renal vein, with perirenal and pararenal varices (Image 2).

Despite reassuring labs and stable hemodynamics, the patient continued to have severe paroxysms of pain. The team repeated her lactate test due to concern for ischemia, but it remained normal. Given her recurrent severe pain and the ultrasound and CT findings, the emergency physician urgently consulted general surgery. The surgeons noted that she was afebrile, hemodynamically stable, and had no lactic acidosis or CT evidence of ischemia; however, her marked abdominal tenderness and breakthrough pain despite multiple high-dose narcotics led them to take her to the operating room for diagnostic exploration.

*CPC-EM Capsule*

What do we already know about this clinical entity?

*Ileocolic intussusception is rare in adults and usually has a pathological lead point; diagnosis is challenging, but emergency department (ED) point-of-care ultrasound (POCUS) can aid early recognition.*

What makes this presentation of disease reportable?

*The case links adult ileocolic intussusception and ischemia to CurQD supplement use and highlights POCUS-driven, expedited surgical management.*

What is the major learning point?

*POCUS can rapidly identify intussusception and prompt urgent surgical involvement even with normal vitals and labs and can be a diagnostic aid with use of new adjunctive therapies.*

How might this improve emergency medicine practice?

*Encourage rapid POCUS use for suspected abdominal pathology, fostering early surgical consultation and targeted imaging, even with normal labs.*

**Image 3:**
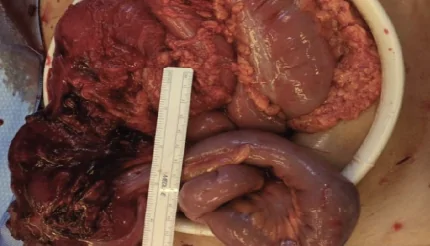
Intraoperative photograph demonstrating an ileocolic intussusception with congested, edematous small bowel and a necrotic segment of colon. A measuring ruler is shown for scale, highlighting the length of ischemic bowel requiring resection.

Laparoscopy revealed the right colon telescoped into the transverse colon to mid-transverse. Due to a lack of reduction with laparoscopic manipulation, the surgeons decided to perform an open laparotomy. The right colon appeared dark purple with patchy deserosalization and thin, near-perforated segments. A right hemicolectomy was performed for ischemic bowel, with end ileostomy creation and a mucus fistula through the same aperture (Image 3). Pathologic analysis demonstrated transmural ischemic necrosis of the right colon without evidence of a mural or intraluminal mass lesion serving as a lead point.^14^–^15^

Postoperatively, the patient’s course was uncomplicated. Pain management included patient-controlled analgesia and, later, oral agents (acetaminophen, oxycodone, and gabapentin; later pregabalin for anxiety modulation). She remained hemodynamically stable, tolerated diet advancement, and had consistent ostomy output. She was discharged on postoperative day 9 with ostomy education and a 28-day course of enoxaparin for venous thromboembolism prophylaxis.

At follow-up, the patient reported good functional recovery. She continued steroid taper for ulcerative colitis and discontinued CurQD following multidisciplinary review of the potential association with her presentation.

## DISCUSSION

Adult intussusception remains an uncommon clinical entity, accounting for only a small percentage of intestinal obstructions. While most cases of intussusception occur in children, the adult form differs markedly in its pathophysiology, presentation, and management. Pediatric intussusception is typically idiopathic and amenable to nonoperative reduction. In contrast, adult cases usually result from an underlying pathology such as a tumor, polyp, or inflammatory process that serves as a lead point.^1^–^4^ This difference makes rapid recognition and surgical intervention critical. However, the nonspecific and intermittent nature of abdominal pain in adults often leads to diagnostic delay.^5^–^8^

Point-of-care ultrasound is a valuable diagnostic tool that can substantially shorten the time to definitive diagnosis and surgical management. Prior reports demonstrate that physician-performed ultrasound can identify the target sign (in short axis) and the “pseudokidney” sign (in long axis) in real time at the bedside, prompting expedited imaging and consultation.^6^,^7^, ^9^,^10^ In our case, bedside POCUS was not only diagnostic but pivotal in advocating for early surgical intervention despite normal vital signs and lab studies. The visual confirmation of a target sign allowed the emergency team to communicate the urgency of the condition to the surgical team, leading to timely exploration and resection before bowel perforation occurred. Similar cases highlight how early POCUS detection in the emergency setting can even decrease time to CT confirmation and operative decision-making, improving outcomes.^4^,^6^

Our patient’s presentation also underscores the importance of maintaining a high index of suspicion for intussusception in patients with inflammatory bowel disease. While ulcerative colitis typically affects the mucosa and submucosa of the colon, chronic inflammation and mucosal edema can create transient areas of dysmotility that predispose to bowel telescoping.^13^–^14^ Additionally, systemic corticosteroids, which our patient was tapering, may alter bowel wall compliance and healing, potentially contributing to ischemic vulnerability.

A particularly notable feature of this case is the possible association with CurQD, a nutraceutical combining curcumin and qing dai (indigo naturalis). CurQD was first used clinically in Israel as early as 2018 and became available as a standardized, commercially marketed nutraceutical protocol in the early 2020s. CurQD has gained attention for its reported anti-inflammatory effects in ulcerative colitis. However, recent post-marketing reports and observational studies have linked qing dai–containing compounds to adverse gastrointestinal outcomes, including right-sided ischemic colitis and rare intussusception events.^15^ The mechanism is not fully understood, but proposed mechanisms include mesenteric vasoconstriction leading to transient regional ischemia, microvascular endothelial injury, and local mucosal irritation from indigo-containing compounds, all of which could theoretically alter segmental motility or precipitate short-lived obstruction. Qing dai has also been associated with crystal deposition and oxidative stress in the intestinal mucosa, suggesting that focal edema or dysmotility may trigger transient intussusception.^15^

This case highlights three major learning points. First, bedside POCUS can be both diagnostic and advocacy-enabling, facilitating rapid multidisciplinary coordination even when lab or hemodynamic data are reassuring. Second, persistent, severe, waxing-and-waning abdominal pain should prompt consideration of intermittent bowel obstruction or intussusception, particularly in patients with inflammatory bowel disease, regardless of lab values or hemodynamic status. Third, as alternative and adjunctive therapies such as CurQD become more widely used, clinicians must remain vigilant about emerging gastrointestinal toxicities and report potential adverse associations to improve post-market safety surveillance.

Ultimately, this case adds to the growing evidence supporting POCUS as a critical adjunct for early diagnosis and advocacy in adult abdominal emergencies. By pairing rapid imaging recognition with clinical judgment, physicians can intervene before irreversible ischemia develops, thereby reducing morbidity and improving patient outcomes.

## CONCLUSION

This case illustrates that point-of-care ultrasound can accelerate diagnosis in adult intussusception, especially when symptoms are intermittent and routine studies are nondefinitive. Early identification of the target sign allowed the care team to proceed quickly with operative management and prevent progression to ischemia. It also highlights the importance of considering use of over-the counter supplements, including newer products such as CurQD, when evaluating unexplained abdominal pathology in patients with inflammatory bowel disease. Combining POCUS with a careful clinical history can support timely and effective intervention in this rare condition.
